# Effect of TheraCal PT and Biodentine on inflammatory cell infiltration and hard tissue formation after pulpotomy in inflamed or healthy rat molars

**DOI:** 10.1007/s00784-025-06372-8

**Published:** 2025-05-17

**Authors:** Tuba Gok, Guzide Cankaya, Yesari Eroksuz, Canan Akdeniz Incili, Suna Karadeniz Saygili

**Affiliations:** 1 Department of Endodontics, School of Dentistry, Firat University, Elazig, 23119 Turkey; 2 Fethi Sekin Oral and Dental Health Center, Elazig, Turkey; 3 Department of Pathology, School of Veterinary Medicine, Firat University, Elazig, Turkey; 4 Department of Histology and Embryology, School of Medicine, Kutahya Health Sciences University, Kutahya, Turkey

**Keywords:** Hard tissue formation, Inflammatory cell infiltration, Rat pulpitis model, Resin-modified calcium silicate cement, Vital pulp therapy

## Abstract

**Objectives:**

TheraCal PT is a novel resin-modified calcium-silicate material introduced for vital pulp therapies. This study aimed to investigate the inflammation and hard tissue formation after pulpotomy treatment with Biodentine and TheraCal PT in inflamed or healthy rat molar teeth.

**Methods:**

This study consisted of six groups (*n* = 12, 72 teeth): negative control-no preparation (NC); positive control (PC); Biodentine-pulpitis teeth (BD-P), TheraCal PT-pulpitis teeth (TPT-P), Biodentine-healthy teeth (BD-H), TheraCal PT-healthy teeth (TPT-P). For PC, BD-P and TPT-P groups, teeth were induced with lipopolysaccharide for 12 h. Pulpotomy procedure was performed with Biodentine and TheraCal PT. Eight weeks later, pulpal inflammatory cell infiltration and hard tissue formation were evaluated by histological analysis. Pearson’s chi-square test was performed.

**Results:**

There were significant differences among groups for inflammation and hard tissue formation (*p* <.05). PC group showed moderate to severe inflammation. Biodentine groups showed lower inflammation scores than TheraCal PT groups. Pulpitis-induced groups showed higher inflammation scores than healthy groups. In Biodentine groups, complete hard tissue formation was higher than incomplete hard tissue formation. TheraCal PT groups showed mostly incomplete or no hard tissue formation. Pulpitis-induced groups showed inferior hard tissue formation scores than healthy groups.

**Conclusions:**

TheraCal PT as a pulp capping material and inflamed pulp as a pre-treatment tooth condition showed inferior results in pulpotomy treatment. Biodentine exhibited favorable inflammatory pulpal responses and thicker hard tissue formation than TheraCal PT.

**Clinical relevance:**

The novel resin-modified calcium-silicate material showed inferior inflammatory cell infiltration and hard tissue formation results in pulpotomy-treated teeth with irreversible pulpitis.

## Introduction

Dental pulp can be exposed to harmful stimuli such as dental caries, trauma and iatrogenic factors. If this situation is not controlled, a series of inflammatory stages may occur, leading to cellular death or necrosis [1]. Pulp inflammation (pulpitis) has been defined as a series of sequential vascular and cellular events mediated by molecular factors in response to microbial, chemical, or, physical (mechanical and thermal) factors [2, 3]. During this dynamic inflammatory process, the dentin-pulp complex offers a series of physiological defense mechanisms included in the term reparative dentinogenesis [4]. It can remain resistant to microbial damage and preserve regenerative potential even when irreversible pulpitis symptoms are present [5].

Today, vital pulp therapies (VPTs), a biologically based minimally invasive therapeutic approach, have gained interest among pulp-preserving treatment practices that include indirect and direct pulp capping to partial and complete pulpotomy [6]. Tissue repair and its extent after VPTs depend on the type of material used, which should stimulate pulp regeneration, induce the formation of a hard tissue barrier following the activation of odontoblasts and show sufficient cytocompatibility against cellular components [7].

Various biomaterials have been proposed and developed over time for VPT procedures (4). Hydraulic calcium silicate-based materials such as mineral trioxide aggregate (MTA; Dentsply-Sirona, Konstanz, Germany) and Biodentine (Septodont, Saint-Maur-Des-Foss es, France) have demonstrated superior ex vivo histological and clinical outcomes in the treatment of exposed pulp [7, 8]. Biodentine and MTA have similar effects on dentin bridge formation [9]. In partial or complete pulpotomies in permanent teeth, Biodentine has been shown to reverse irreversible pulpitis when used as a dressing on the pulp [10]. In addition to their superior properties on the vital pulp, hydraulic calcium silicate-based materials also have some limitations. The setting time of MTA is long and not optimized, it is difficult to handle, the strength is not adequate and it causes discoloration of teeth [8]. The initial phase of Biodentine’s setting reaction is relatively long, approximately 12 min, while final maturation is approximately 14–30 days [11]. To overcome hydraulic calcium silicate-based materials’ limitations, light curable resin-modified calcium silicate-based materials have been developed [12].

The first marketed resin-modified calcium silicate-based material TheraCal LC (Bisco Inc, Schaumburg, IL) has been extensively studied in vitro, in vivo, and clinically and has shown controversial findings. Consequently, various authors have recommended its clinical performance be limited to indirect pulp capping [13, 14]. More recently, TheraCal PT (Bisco Inc), a dual-cured resin-modified calcium silicate material, has been marketed by improving the chemical properties of TheraCal LC to overcome the adverse effects of unpolymerized residual monomers [15]. According to its manufacturer, TheraCal PT preserves the viability of pulp tissue and is indicated for pulpotomy, indirect and direct pulp capping therapies [16]. However, in-vitro stem cell studies with TheraCal PT have revealed conflicting results [16–19]. Although promising results have been reported, with cytocompatibility and bioactivity levels of TheraCal PT being similar to Biodentine [17] and white ProRoot MTA [18] (Dentsply Tulsa, OK, USA), some studies have demonstrated lower cell viability [16] and higher cytotoxicity than other calcium silicate cements [19].

The rat molar can mimic the human molar anatomically, histologically, biologically and physiologically, therefore, the rat molar is a good model to evaluate pulpal tissue reactivity in the experimental pulpitis model [20]. It has been shown that calcium silicate-based cements induce dentin bridge formation in a rat mechanical dental injury model [21]. Additionally, odontoblast-like cells of rat molar teeth have been reported to produce reparative dentin within 2 weeks following stimulation [22].

To our knowledge, TheraCal PT has not been previously investigated in terms of healing and hard tissue formation in inflamed pulps by creating an experimental pulpitis model in pulpotomy treatments. Moreover, the success of vital pulp treatment with resin-modified calcium silicate-based material in both healthy and inflamed pulps has not been compared in an in vivo model. Therefore, this study aimed to investigate the inflammatory cell infiltration of the pulp and hard tissue formation after pulpotomy treatment with two different calcium silicate-based materials (Biodentine and TheraCal PT) in inflamed or healthy rat molar teeth. The null hypothesis was that there would be no differences in pulpal inflammation or hard tissue formation according to the cement type and pulp status before the treatment.

## Materials and methods

The study was reviewed and approved by the Firat University Institutional Local Ethics Committee for Animal Experiments (experimental protocol number 2022/07).

### Sample size calculation

The sample size was calculated based on a previous study [23]. The G*Power 3.1.9.7 software (Heinrich Heine University, D€usseldorf, Germany) was used to determine sample size and a minimum of 9 samples per group was determined. Anticipating that rats or filled teeth might be lost during the follow-up period, the number of samples per group was determined as 12.

All experiments were performed with 60 male Sprague-Dawley rats (6 groups, 72 teeth; for the negative control group teeth, the upper left first molars, which were in the opposite quadrant of the maxilla of the rats in the Biodentine healthy group, were used), 8-week-old and weighing 250–300 g. Rats were under standard controlled conditions, kept in polyethylene cages at controlled temperature (23 ± 2 ℃) and humidity (55% ± 10%) with a 12-hour light/12-hour dark cycle. They were fed with water and a pelleted rat diet ad libitum. There was an experienced veterinary technician responsible for the care of the rats, who followed all feeding recommendations. The rats were acclimated for a week to adapt to their new environment and to reduce stress levels. Then they were randomly divided into their groups (*n* = 12).

#### Negative control group (NC)

No procedure was performed.

#### Positive control group (PC)

Lipopolysaccharide (LPS)-treated pulp (12 h), pulpotomy procedure and permanent restoration with glass ionomer cement.

#### Pulpotomy treatment with Biodentine in teeth with pulpitis (BD-P)

LPS-treated pulp (12 h), pulpotomy procedure, placement of Biodentine and permanent restoration with resin composite.

#### Pulpotomy treatment with TheraCal PT in teeth with pulpitis (TPT-P)

LPS-treated pulp (12 h), pulpotomy procedure, placement of TheraCal PT and permanent restoration with resin composite.

#### Pulpotomy treatment with Biodentine in healthy teeth (BD-H)

Pulpotomy procedure, placement of Biodentine and permanent restoration with resin composite.

#### Pulpotomy treatment with TheraCal PT in healthy teeth (TPT-H)

Pulpotomy procedure, placement of TheraCal PT and permanent restoration with resin composite.

### Experimental pulpitis model

**Fig. 1:**
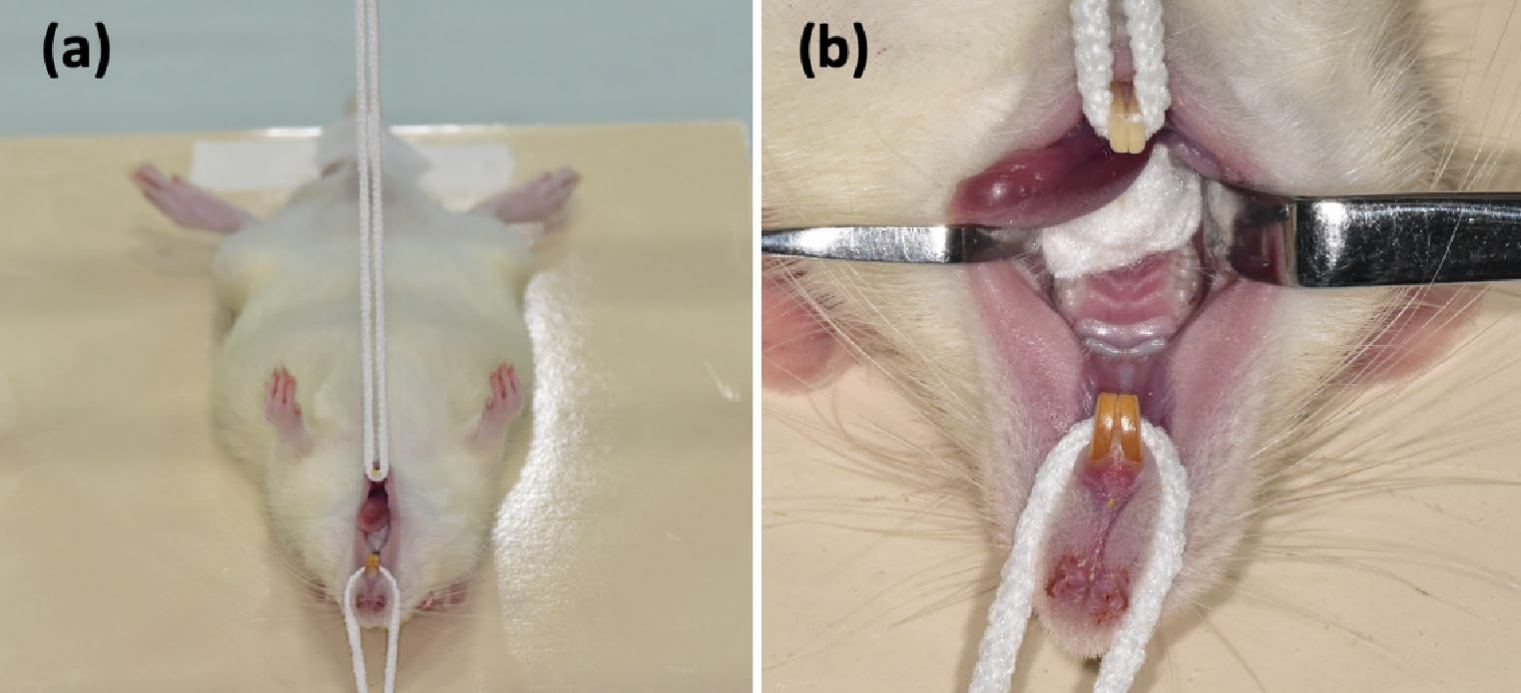
Representative image of the pre-treatment 8-week-old rat placed in dorsal decubitus position on the custom-made table (**a**). The use of retractors for better visualization (**b**)

**Fig. 2:**
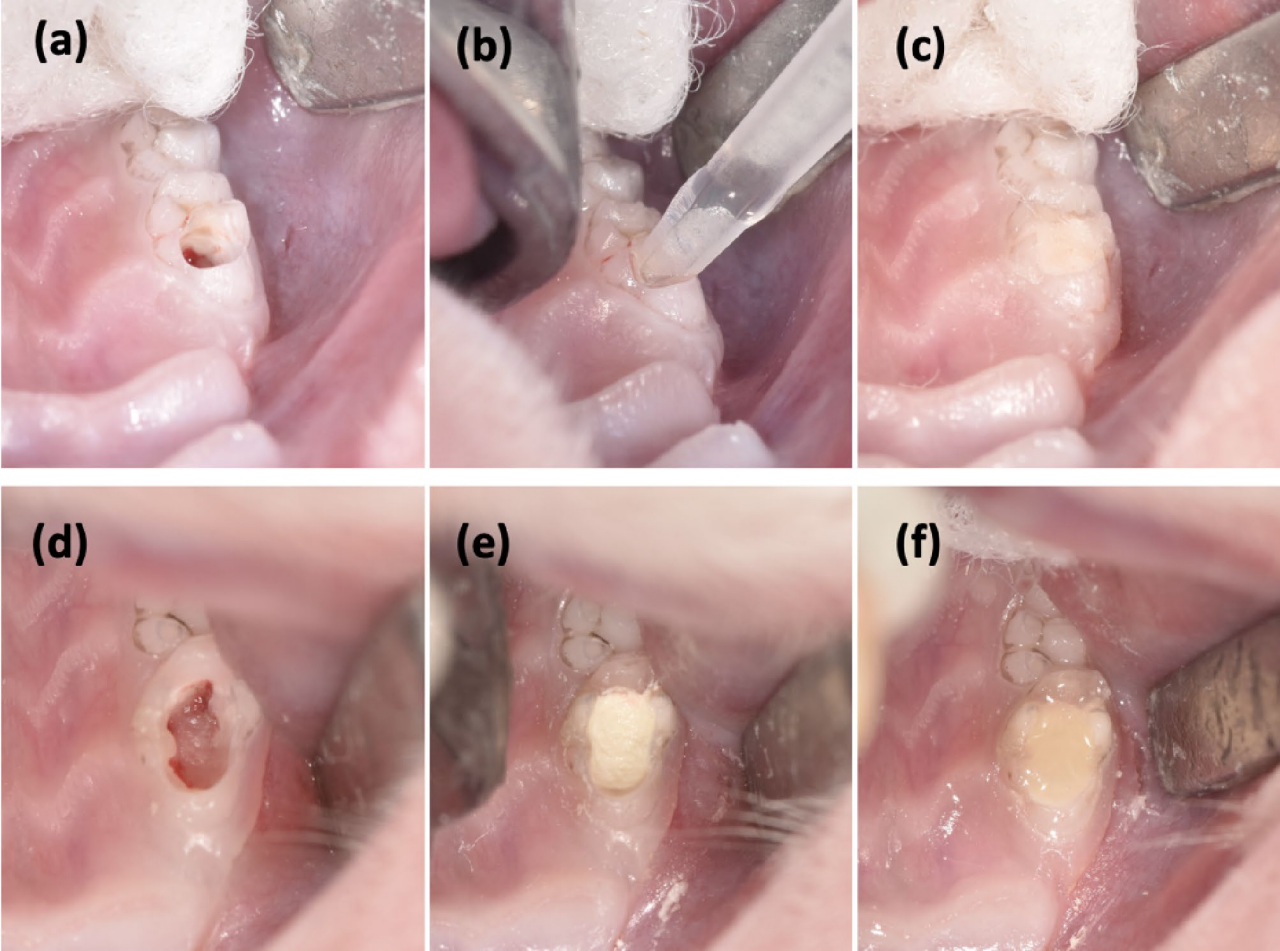
Representative images of the clinical procedure of LPS induction (**a**-**c**) and pulpotomy treatment (**d**-**f**). (**a**) Pulp exposure with a diamond bur. (**b**) Applying of LPS. (**c**) Temporarily sealing the cavity with glass ionomer cement for 12 h. (**d**) Removal of coronal pulp tissue after cavity preparation. (**e**) Calcium silicate cement placement onto the exposed pulp tissue. (**f**) Permanent restoration with resin composite

Rats were anesthetized intraperitoneally with 5 mg/kg xylazine (Rompun; Bayer, Istanbul, Turkey) and 45 mg/kg ketamine (Ketasol 10%; Richter Pharma Ag, Wels, Austria). To achieve successful hemostasis, infiltrative anesthesia was performed on the tooth with 2% lidocaine (Lidocaine HCL 2%; Osel, Istanbul, Turkey) containing 1:100 000 epinephrine (4.4 mg kg − 1 dose − 1) [24]. The lower limbs of the rats were affixed to a custom-made surgical table and placed in the dorsal decubitus position (Fig. 1a). Retractors were used for better visualization (Fig. 1b). Before the preparation teeth were disinfected with 2% chlorhexidine gluconate (Bisco, Schaumburg, IL, USA). Pulp exposure was achieved in the upper right first molar with a sterile diamond bur (0.8 mm diameter) (Komet group, Lemgo, Germany) at 3,500 rpm under water cooling with sterile distilled water (Fig. 2a). Bleeding control was achieved by applying gentle pressure to the cavity of sterile cotton pellets moistened with 0.5% NaOCl for 1–3 min. Experimental pulpitis was performed by applying five microliters of LPS from Escherichia coli O111:B4 (Sigma Chemical, Darmstadt, Germany) diluted in phosphate-buffered saline to a final concentration of 10 mg/mL onto the injured pulp (Fig. 2b), as described by Renard et al. [25]. The cavities were temporarily sealed with glass ionomer cement (R&D Series Nova Glass-F; Imicryl, Konya, Turkey) (Fig. 2c). All procedures were performed under magnification (3.5X; Zumax Medical Co. Ltd., Suzhou, China).

### Pulpotomy procedure

Twelve hours after LPS application (in pulpitis model created groups; PC, BD-P, TPT-P), the temporary fillings and dentin tissue were removed and total pulpotomy was performed (Fig. 2d). In healthy teeth groups, dentin tissue was directly removed and total pulpotomy was performed up to the orifices under the same anesthesia conditions. After the bleeding control (as mentioned above), for Biodentine groups; Biodentine was placed on the cavity floor with the MAP-System (Produits Dentaires SA, Vevey, Switzerland) and lightly condensed with a plugger and a setting time (12 min) was allowed (Fig. 2e). For TheraCal PT groups; TheraCal PT was placed on the cavity floor with the mixer tip following the manufacturer’s instructions and ensured good adaptation to the cavity walls and margins, then light cured for 10 s (Woodpecker LED.B; Keju Medical Products, Foshan, China). The permanent restorations were completed with resin composite (Arabesk; Voco, Cuxhaven, Germany) (Fig. 2f). In the positive control group, the glass ionomer cement (Nova Glass-F) was applied as a permanent restoration after the pulpotomy procedure. To minimize occlusal forces, occlusal reduction was performed on the opposing tooth. In the post-treatment period, the health status of the rats (behavior, body weight, skin and hair changes, food and water consumption, urine and defecation) was checked daily. After 8 weeks of follow-up, the rats were sacrificed using intraperitoneal injections with an overdose of anesthetic solution.

### Histological examination

The mandibles were dissected and the teeth were removed. The samples were fixed immediately in 10% formalin for at least 48 h and then decalcified in 10% ethylenediaminetetraacetic acid (EDTA) for 6 weeks. The flexibility of the teeth was checked and samples were cleared in xylene and embedded in paraffin. 5 μm thick serial section (5 sections for each sample) were cut in the transverse plane by using a microtome, followed by deparaffinization and rehydration. The largest section containing the maximum area of the injury site in each sample was selected for histological analysis.

The sections were stained with hematoxylin and eosin (H&E) and observed under 4× to 20× magnification with a light microscope (AxioVision; Zeiss, Oberkochen, Germany). Two experienced observers blinded to the groups examined all sections. They evaluated the hard tissue formation and inflammatory cell infiltration of the pulp with a scoring system as reported in previous studies [26, 27] (Table 1). In sections where there was disagreement, observers reached a consensus after negotiation.

**Table 1:** Assessment criteria for the inflammatory cell infiltration and hard tissue formation after pulpotomy

Score	Definition
	**Inflammatory Cell Infiltration**
0	None: absence or few scattering inflammatory cells
1	Mild: scattered inflammatory cells in the pulp tissue adjacent to the pulp exposure
2	Moderate: inflammatory cells with small focal groupings in the pulp tissue adjacent to the pulp exposure
3	Severe: extensive inflammatory cell infiltration in the pulp tissue adjacent to the pulp exposure or necrotic
	**Hard Tissue Formation**
0	None: no hard tissue deposition
1	Incomplete: moderate hard tissue formation and discontinuous dentin bridge
2	Complete: heavy hard tissue deposition and continuous dentin bridge

### Data presentation and statistical analysis

Pearson’s chi-square test was conducted to compare the inflammatory cell infiltration and hard tissue formation scores of the histologic sections of groups. The statistical analyses were performed using SPSS for Windows, Version 26.0 (IBM Corp., Armonk, NY, USA) and *P* <.05 indicated statistical significance.

## Results

The general health status of the rats remained stable throughout the 8-week experiment. At the end of the experiment, no significant difference was observed between the groups regarding average body weight (data can be shared if requested).

**Fig. 3:**
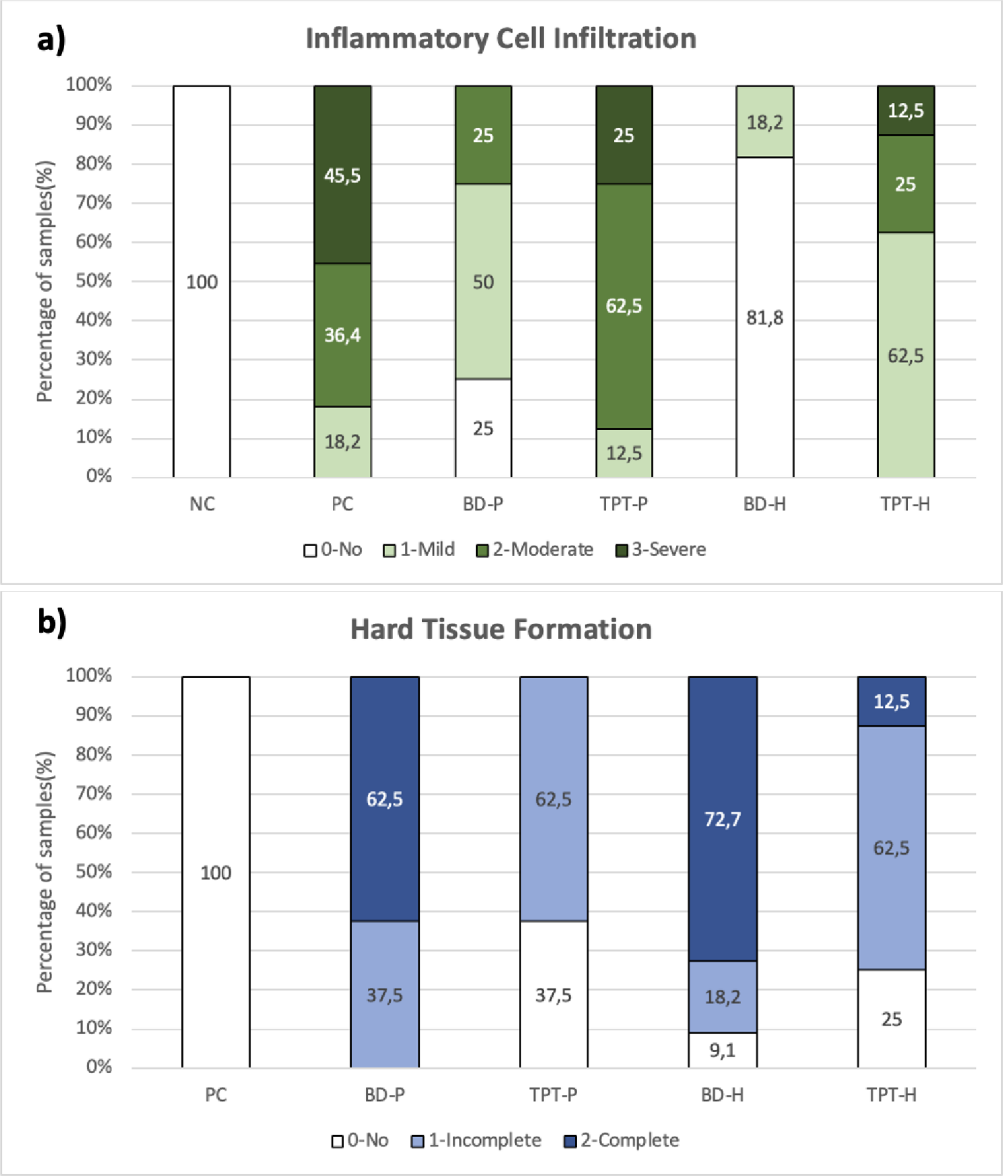
Inflammatory cell infiltration (**a**) and hard tissue formation scores (**b**) graded based on Table 1

Fifteen teeth were excluded during the specimen preparation for histologic analysis and the reason for filling failure. The fillings were relatively large due to the total pulpotomy cavity, therefore fillings failed in some teeth and crowns fractured in some due to the rats being fed a hard pelleted rat diet. The remaining 57 teeth with intact fillings were analyzed. The findings of both the inflammatory cell infiltration and hard tissue formation have been presented in Table 2; Fig. 3a, b.

**Table 2:** Summary of the inflammatory cell infiltration and hard tissue formation scores

	Inflammatory cell infiltration scores *n*(%)				Hard tissue formation scores *n*(%)		
Groups	0	1	2	3	0	1	2
NC	11(100)	0(0)	0(0)	0(0)	-	-	-
PC	0(0)	2(18.2)	4(36.4)	5(45.5)	11(100)	0(0)	0(0)
BD-P	2(25)	4(50)	2(25)	0(0)	0(0)	3(37.5)	5(62.5)
TPT-P	0(0)	2(12.5)	5(62.5)	1(25)	3(37.5)	5(62.5)	0(0)
BD-H	9(81.8)	2(18.2)	0(0)	0(0)	1(9.1)	2(18.2)	8(72.7)
TPT-H	0(0)	5(62.5)	2(25)	1(12.5)	2(25)	5(62.5)	1(12.5)
***P*** **value**	< 0.001*				< 0.001*		

*Asterisk indicates a significant difference (*P* <.05)

### Pulpal inflammatory response

**Fig. 4:**
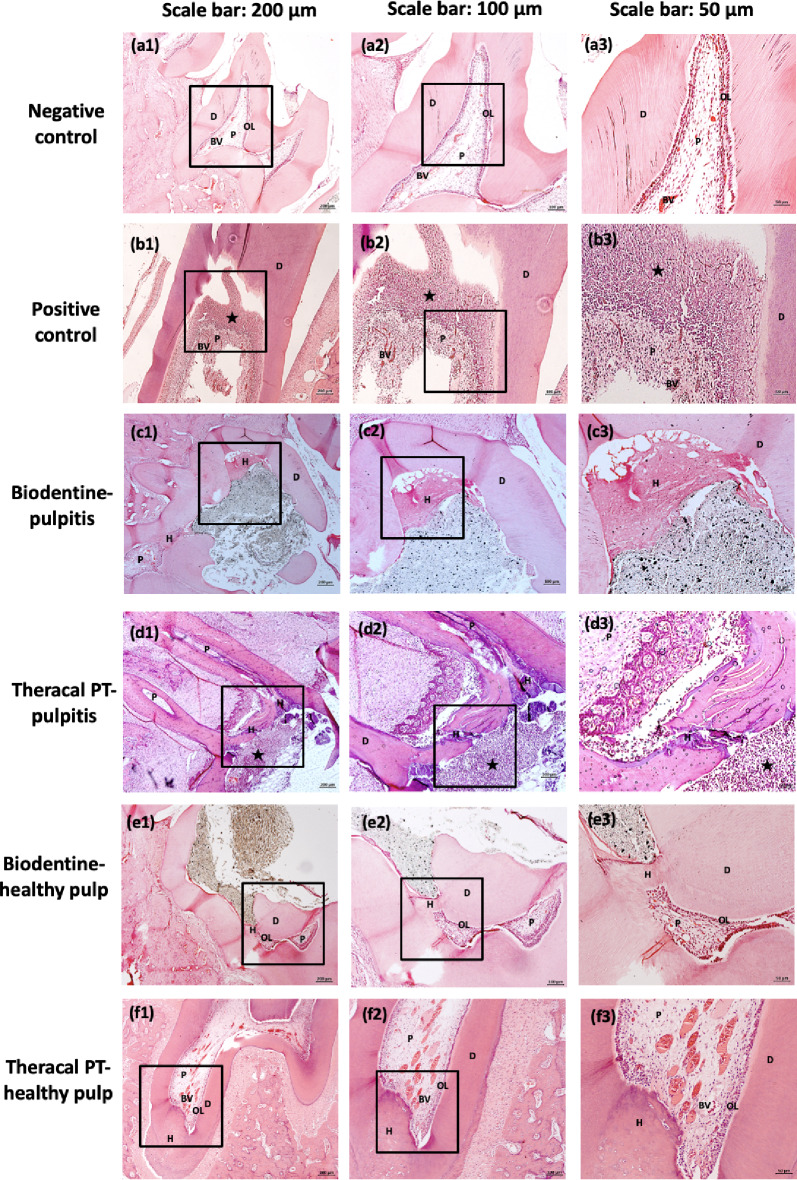
Representative histological images of rat molar teeth 8 weeks after pulpotomy treatment. Hematoxylin-eosin (H&E) evaluation was observed in (**a1**-**a3**) negative control group, (**b1**-**b3**) positive control group, (**c1**-**c3**) Biodentine with pulpitis treated group, (**d1**-**d3**) TheraCal with pulpitis treated group, (**e1**-**e3**) Biodentine with healthy pulp treated group, (**f1**-**f3**) TheraCal PT with healthy pulp treated group. (**a1**-**f1**) present low magnification (4X), (**a2**-**f2**) present higher magnification of the areas (inset rectangles) in **a1**–**f1** (10X) and **a3**-**f3** present higher magnification of the areas (inset rectangles) in **a2**–**f2** (20X). (**a1**-**a3**) represent no inflammatory cell infiltration and a healthy odontoblastic layer in the negative control group. (**b1**-**b3**) represent intense inflammatory infiltration and no hard tissue formation in the positive control group. (**c1**-**c3**) represent no inflammatory cell infiltration and thick and complete hard tissue formation in the BD-P group. (**d1**-**d3**) represent incomplete hard tissue formation with moderate inflammatory cell infiltration in the TPT-P group. (**e1**-**e3**) represent thick and complete hard tissue formation and no inflammatory cell infiltration in the BD-H group. (**f1**-**f3**) represent incomplete hard tissue formation with mild inflammatory cell infiltration in the TPT-H group. BV, blood vessel; D, dentin; H, hard tissue formation; OL, odontoblastic layer; P, pulp; *(asterisk), indicates inflammatory cell infiltration

Significant differences were observed among the inflammation scores of the groups (*P* <.05). HE staining revealed that the NC group had no pathological changes in the dentine/predentine/odontoblast complex or inflammatory cell responses (Fig. 4a1-a3). PC group showed mostly mild to severe infiltration scores (36.4%, 45.5%). Inflammation was present in all specimens of the PC group (Fig. 4b1-b3). Biodentine groups showed lower inflammation scores than TheraCal PT groups, moreover, complete recovery from inflammation was evident in 25%, and 81.8% of the BD-P and BD-H samples, respectively (Fig. 4c1-c3, 4e1-e3). Depending on the presence of inflammation before pulpotomy treatment, inflamed pulp groups showed more inflammatory cell infiltration scores than non-inflamed pulp groups; while the materials were evaluated within themselves. The BD-P group showed more mild or moderate results than the BD-H group (Fig. 4c1-c3, e1-e3) and the TPT-P group showed more moderate or severe scores than the TPT-H group, (Fig. 4d1-d3, f1-f3). Inflammation was present mild to severe in all specimens of the TheraCal PT groups.

### Hard tissue formation

Significant differences were observed among the hard tissue formation of the groups (*P* <.05). No signs of hard tissue formation were present for the PC group (Fig. 4b1-b3). Complete and thicker hard tissue formation was evident in Biodentine groups; (62.5% - BD-P, and 72.7% - BD-H groups) (Fig. 4c1-c3, 4e1-e3). TheraCal PT groups showed 62.5% incomplete hard tissue formations and no hard tissue formation 37.5% in TPT-P and 25% in TPT-H groups (Fig. 4d1-d3, f1-f3). When the same material is considered, hard tissue formation scores in healthy groups were superior compared to pulpitis groups.

## Discussion

With the development of new biocompatible pulp capping materials, VPTs are now considered an alternative to root canal procedures as they show long-term success by promoting pulp tissue regeneration [28]. This study investigated the inflammatory cell responses of the pulp and hard tissue formation after pulpotomy treatment with two different calcium silicate-based materials (Biodentine and TheraCal PT) in rat molar teeth with inflamed or healthy pulp. Significant differences were found in the pulpal inflammatory response and hard tissue formation among the groups (*P* <.05); therefore, the null hypothesis was rejected.

Animal studies have been conducted using pulp-capping materials to investigate the effects on hard tissue formation and inflammation [20, 29]. It was stated that rat molars are suitable for testing pulp capping materials [20]; even if it is not entirely clinically relevant, it provides a mean to study the cellular and molecular mechanisms that occur during dental pulp repair [30]. Rat molars have a larger pulp chamber and limited growth compared to incisors, they have immunocompetent cells with a distribution comparable to human [31], therefore molar teeth were used in our study.

In this study, the procedures were performed under general anesthesia. As in previous studies, local infiltration anesthesia was applied to the surgical side to ensure successful hemostasis before removal of the coronal pulp [24, 32]. Split mouth design was not used in experimental groups. In case of possible tooth pain during one-sided filling, the benefit of the rat was considered to allow the rat to chew and feed from the opposite jaw.

Alternatives include exposing the pulp to the oral environment [29] or applying LPS within the cavity to create a pulpitis model [32]. In this study, the acute inflammation model created using LPS is a widely used method in the simulation of endodontic infections [25, 33]. Application of LPS in a controlled manner ensures reliable and reproducible results [34]. Recently, the levels of various cytokines were shown to be equally up-regulated 6–24 h after LPS-induced inflammation in rat molars. They suggested that short stimulation times are sufficient to observe the pro-inflammatory cytokine levels [35]. Additionally, histological and microscopic findings have shown that LPS application triggers pulpal inflammation, a significant inflammatory response occurs, and this response reaches peak values after 12 h [32]. Therefore, pulpotomy was performed 12 h after induction in the groups induced with LPS in this study.

In most animal studies evaluating the effects of pulp capping materials on hard tissue formation and inflammation, healthy teeth were used [20, 29, 36]. Few studies have been conducted to create an experimental pulpitis model [32, 37]. In an animal study, healthy rat teeth were used with a direct pulp capping model with TheraCal PT, TheraCal LC and ProRoot MTA [18]. Since vital pulp treatments are generally applied to pulp diseased due to caries, healthy tooth models do not accurately represent clinical pulp pathology. However, situations such as acute dental trauma cases or iatrogenic pulp perforations in healthy teeth (during prosthetic tooth preparations) may also be encountered clinically, and in such cases, material selection is important. Therefore, the experimental pulpitis model and healthy teeth groups were used in our study, considering both clinical conditions.

In the methodology, the positive control group was restored entirely with glass ionomer cement, whereas the experimental groups received composite resin restorations over the tested biomaterials (BD and TPT). The rationale for using glass ionomer cement in the positive control group was to evaluate pulpal responses without the tested biomaterials, following pulpitis induction with LPS. Although previous studies have reported more favorable outcomes for glass ionomer cement than for composite in terms of inflammation and cell survival on pulp tissues or fibroblasts [38, 39], their use is generally limited to indirect pulp capping procedures [40] due to the potential for an inflammatory response when in direct contact with connective tissue [41]. In our study, exposing the pulp to the oral environment without any restoration was not feasible due to ethical and methodological constraints. Glass ionomer cement was used for the positive control group as it is less cytotoxic than composite [39], although not as biocompatible as calcium silicate-based materials [42]. It should be emphasized that this situation was a limitation of the study. Glass ionomer cements have been reported to tend to crack or microleakage and demonstrate limited durability, potentially resulting in restoration failure for long-term applications [43]. However, in our study, the experimental period was 8 weeks, and when the teeth were examined after removal, the glass ionomer restorations remained intact and functionally stable.

The effect of pretreatment pulpal inflammation on the tissue response after the pulpotomy procedure is still a concern [5]. In the absence of bacteria, the natural healing process of pulp tissue continues the formation of reparative dentin [44] and pulp capping materials modulate the healing process [27]. In our study, lower scores of inflammatory cell infiltration were observed in the Biodentine and TheraCal PT groups with healthy pulps compared to the Biodentine and TheraCal PT groups with inflamed pulps. Hard tissue formation was also superior in healthy pulp groups. It was reported that a low-pH environment damaged the mechanical properties of pulp-capping materials in a study that mimicked the clinical environment of pulp inflammation [45]. Although it is unclear to what extent the mechanical properties of the affected materials affect their role in modulating the healing process of the pulp or hard tissue formation, they may have led to less successful findings in the inflamed pulp. Santos et al. assessed the influence of preoperative pulp inflammation in the outcome of full pulpotomy using a dog model [46]. Contrary to our findings, no significant differences were observed between normal and inflamed pulp, regardless of the evaluated histologic parameters. In their study, pulpal inflammation was achieved by exposing dentin tissue to the oral environment, whereas in our study, LPS was applied directly to the pulp. The difference in findings may be due to methodological differences.

According to the present study, Biodentine groups showed superior results than TheraCal PT groups, which showed lower inflammatory cell infiltration and thicker hard tissue formation in healthy and inflamed teeth groups. To our knowledge, there is no in vivo study comparing Biodentine with TheraCal PT therefore, these results cannot be compared with other studies. TheraCal PT was evaluated in two studies with other calcium silicate cements that have similar success rates to Biodentine [18, 47]. Park et al. compared TheraCal PT and ProRoot MTA in a rat direct pulp capping model with healthy pulp [18]. ProRoot MTA showed a higher no-inflammation score (87.5%) than TheraCal PT, similar to our healthy pulp group findings. Unlike our study, moderate or severe inflammatory cell response showed similar results with TheraCal PT (25%). This may be due to the difference in experimental time between studies (4–8 weeks) and the difference in VPT (direct pulp capping-pulpotomy). In another study, TheraCal PT, MTA Angelus and NeoMTA (NuSmile Avalon Biomed, Bradenton, FL, USA) were compared in rat incisor teeth with healthy pulp [47]. After 45 days, all groups showed similar findings of mild inflammation. It is well-known that rodent incisors have a high healing capacity due to their continuously erupting process [25]. Therefore, using incisor or molar teeth may cause differences with our findings.

The materials used in VPTs should have anti-inflammatory properties and play a role in the proliferation, migration and mineralization of dental pulp stem cells [48]. In some of the in vitro stem cell studies conducted for this purpose, although TheraCal PT showed a similar success rate with other calcium silicate cements [17, 18], most of them showed inferior results [16, 19, 49]. Sanz et al. [17] reported that the cell survival percentage of undiluted TheraCal PT was less than Biodentine at 72 h (91.2% and 86.7%, respectively). Rodriguez-Lozano et al. [16] stated that TheraCal PT had significantly less cell survival than MTA. Kuden et al. [19] reported that there were no viable cells left in the TheraCal PT group by day 7 and the authors showed the time-dependent monomer release of TheraCal PT as the reason for this and stated that a significant amount of TEGDMA (triethylene glycol dimethacrylate) was released on the 3rd and 7th days of TheraCal PT. Although the behavior of materials tested in vitro can potentially be affected by several external factors in the clinical environment [50], these findings support the inferior findings obtained with TheraCal PT in our study.

Materials used in VPT procedures must have the ability to induce superficial mineralization of viable pulp tissue to form a calcified bridge. Studies have shown materials such as MTA and Biodentine to have positive clinical results associated with a mild inflammatory response [51, 52]. It has also been suggested that mild inflammation is associated with thicker and more continuous dentin bridge formation [52]. Similarly, complete hard tissue formation values and mild inflammation scores in our study were higher in the Biodentine groups. Therefore, the observation of higher levels of inflammatory responses in the TheraCal PT groups may have resulted in lower or absent hard tissue formation scores. Ca2 + ion release induces pyrophosphatase activity, thus preserving dentin mineralization and improving the dentin bridge [53]. In previous studies it was stated that Ca2 + release in TheraCal PT is lower than in Biodentine [17, 49] this may also explain the findings of inferior hard tissue formation in TheraCal PT groups.

This study has limitations. Pulpotomy treatment on a rat molar was challenging due to its small size and pulpotomy as a procedure for evaluation of a tiny area of pulp tissue at a canal orifice is also challenging. Rats’ oral environment and plaque structure differ from humans [47]. However, rat models are widely used in research as a preliminary approach before clinical use in evaluating new materials to mimic human-like biological properties and provide reliable responses [54]. Most of the studies used healthy teeth for comparing pulp capping materials, using both inflamed and healthy pulp groups was the strength of this study. Future studies need to be conducted in more clinically similar situations, such as carious teeth with inflamed pulps, and long-time follow-up.

## Conclusion

Within the limitation of the current study, the pre-existing inflammation of the pulp before treatment negatively affected the results of pulpotomy treatment. TheraCal PT showed inferior results in terms of inflammatory cell infiltration and hard tissue formation. Biodentine exhibited favorable inflammatory pulpal responses and thicker hard tissue formation. To properly evaluate the clinical potential of Theracal PT, further investigations with a long period should be conducted on its use as a pulp capping material in animal models.

## Data Availability

No datasets were generated or analysed during the current study.
